# Prediction-driven matched molecular pairs to interpret QSARs and aid the molecular optimization process

**DOI:** 10.1186/s13321-014-0048-0

**Published:** 2014-12-11

**Authors:** Yurii Sushko, Sergii Novotarskyi, Robert Körner, Joachim Vogt, Ahmed Abdelaziz, Igor V Tetko

**Affiliations:** eADMET GmbH, Lichtenbergstraße 8, D-85748 Garching, Munich Germany; Helmholtz-Zentrum München - German Research Centre for Environmental Health (GmbH), Institute of Structural Biology, Ingolstädter Landstraße 1, D-85764 Neuherberg, Germany; A.M. Butlerov Institute of Chemistry, Kazan Federal University, Kremlyovskaya St. 18, 420008 Kazan, Russia

**Keywords:** MMP, Matched molecular pairs, QSAR, Interpretation, Molecule optimization, Medicinal chemistry, Inverse QSAR, OCHEM, Online chemical modelling environment

## Abstract

**Background:**

QSAR is an established and powerful method for cheap *in silico* assessment of physicochemical properties and biological activities of chemical compounds. However, QSAR models are rather complex mathematical constructs that cannot easily be interpreted. Medicinal chemists would benefit from practical guidance regarding which molecules to synthesize.

Another possible approach is analysis of pairs of very similar molecules, so-called matched molecular pairs (MMPs). Such an approach allows identification of molecular transformations that affect particular activities (e.g. toxicity). In contrast to QSAR, chemical interpretation of these transformations is straightforward. Furthermore, such transformations can give medicinal chemists useful hints for the hit-to-lead optimization process.

**Results:**

The current study suggests a combination of QSAR and MMP approaches by finding MMP transformations based on QSAR predictions for large chemical datasets. The study shows that such an approach, referred to as prediction-driven MMP analysis, is a useful tool for medicinal chemists, allowing identification of large numbers of “interesting” transformations that can be used to drive the molecular optimization process. All the methodological developments have been implemented as software products available online as part of OCHEM (http://ochem.eu/).

**Conclusions:**

The prediction-driven MMPs methodology was exemplified by two use cases: modelling of aquatic toxicity and CYP3A4 inhibition. This approach helped us to interpret QSAR models and allowed identification of a number of “significant” molecular transformations that affect the desired properties. This can facilitate drug design as a part of molecular optimization process.

**Graphical Abstract:**
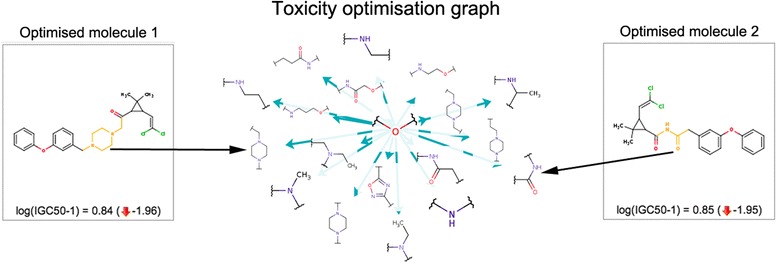
Molecular matched pairs and transformation graphs facilitate interpretable molecular optimisation process.

## Background

Quantitative Structure Activity Relationships (QSAR) have proven to be a powerful technique for prediction of biological activities and physicochemical properties. QSAR models can be helpful in drug design, ecological hazard assessment and in the chemical industry. The properties predicted by QSARs vary from solubility and melting point to toxicity, biological potency and possible side effects [1-3].

One of the issues with QSAR models is their poor interpretability. While interpretation of simple linear regressions can be straightforward, the most powerful algorithms like neural networks are similar to “black boxes”, which provide predictions that cannot be easily interpreted. This undermines trust in such predictions and prevents the creation of an “action plan” by a decision maker, for example a medicinal chemist. If a compound is predicted to be toxic, what are the causal factors for its toxicity? How can it be made non-toxic? Such “black-box” model types are poorly suited to address these crucial practical questions.

The QSAR interpretation problem has not escaped the notice of regulatory bodies. Thus, “mechanistic interpretation” is one of the principles of the OECD for acceptable QSAR predictive models for regulatory purposes [4].

**Figure 1:**
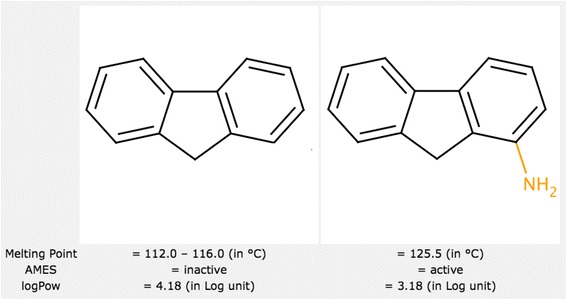
**Four typical examples of matched molecular pairs (MMPs).**

A recently suggested approach for the analysis of chemical datasets uses pairs of compounds that differ by a small single point change only. Such pairs are referred to as matched molecular pairs or MMPs [5,6]. An analytical approach that deals with MMPs is matched molecular pairs analysis (MMPA). A number of MMPs are given as examples in Figure 1.

**Figure 2:**
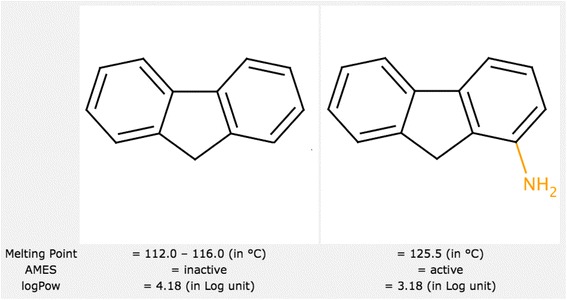
**Activity cliff example.** A molecule inactive according to the Ames test becomes active after a minor structural change. Activity cliffs represent interesting cases for activity interpretation.

Analysis of pairs of molecules with only minor localized differences can be very useful for understanding the mechanism of action. A significant change of activity following only a minor structural modification (known as an “activity cliff”) can give additional insight (see Figure 2). Furthermore, using simple statistical analysis molecular pairs can be grouped by transformations, allowing the identification of transformations that affect properties of interest.

In the scientific literature [7], MMP analysis has focused mostly on analysis of experimental data and on trying to identify rules that affect activities of interest. Such analyses are not directly related to QSAR modelling, representing rather a complementary approach. The current study aims to merge the worlds of QSAR- and MMP-based analysis by introducing the concept of *prediction-driven MMPs*.

MMP analysis has a practical goal: molecular transformations can help medicinal chemists to drive the molecular optimization process. This task is not directly achievable through plain QSAR analysis. A QSAR model provides predictions but does not explicitly identify how a structure should be changed in order to achieve the desired improvements (such as reducing toxicity, enhancing activity or improving the ADME profile). This problem is sometimes referred as inverse QSAR. This study investigates how prediction-driven MMP rules can guide the molecular optimization process.

The study is not limited to theoretical developments. We also provide software implementation of all the analytical utilities – including identification of MMPs, statistical analysis, visualization and interpretation utilities – and tight integration with the database of experimental data and a QSAR modelling framework. This study represents the “tip of an iceberg”: a molecule optimizer utility that can be used by medicinal chemists to optimize molecules with regard to endpoints such as mutagenicity, CYP inhibition, environmental toxicity, solubility and lipophilicity. All utilities have been integrated into the Online Chemical Modelling Environment and are freely available to the academic community online at http://ochem.eu/.

## Results and Discussion

In this section, we apply the prediction-driven matched molecular pairs analysis for the QSAR models to the two endpoints mentioned earlier: aquatic toxicity and CYP3A4 inhibition. For each endpoint, we demonstrate the additional knowledge gained from the prediction-driven transformations and the practical value of such knowledge for the molecular optimization procedure.

Significant transformations were defined as those with a statistical significance of at least p <0.05. We used the Holm-Bonferroni method [8] to reduce false positives caused by the multiple comparisons problem. The statistical significance (p-value) was calculated according to formulae (1) and (2) described in the methodology section. To avoid highly dissimilar matched pairs, we considered only pairs of molecules with a Tanimoto similarity of at least 50% calculated using ECFP fingerprints [9] for the analysis.

A summary of the significant transformations identified using experimental data as well as the prediction-driven analysis is given in Table 1. After applying the Holm-Bonferroni method, approximately half of the transformations were discarded. Below, we provide a detailed analysis of all endpoints and their respective models.

**Table 1:** **The number of significant transformations identified using experimental and predicted datasets**

**Endpoint**	**Significant transformations**		
	**Experimental-based**	**Prediction-based**	
		**EINECS**	**ChemDiv**
Aquatic toxicity	119	1552	5301
Ames test	132	442	4397

Clearly, prediction-based analysis provides significantly more transformations.

**Figure 3:**
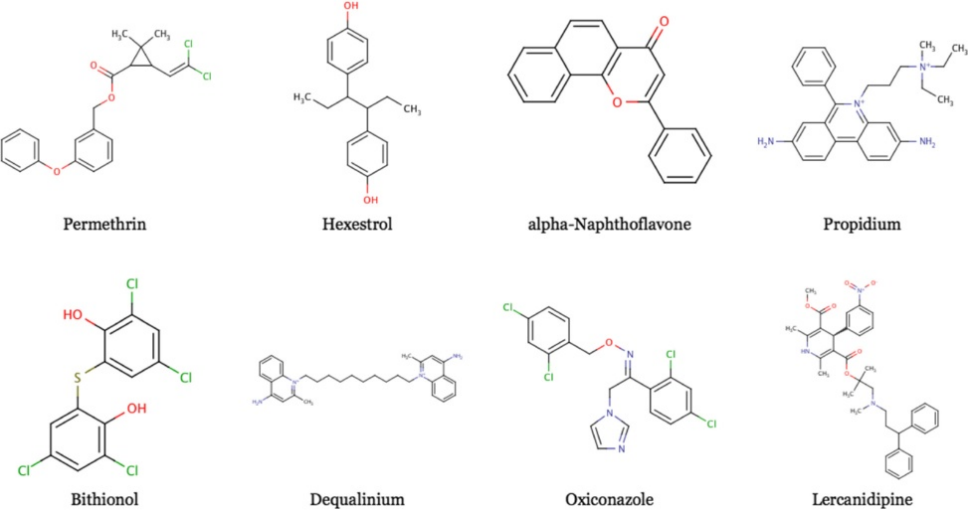
**Molecules transformed within the scope of this study.** All eight molecules were selected from the DrugBank database.

The target molecules used as examples of transformation-driven optimization are shown in Figure 3. These comprise several representative molecules from the DrugBank database [10,11]. Some are marketed drugs, while others are experimental molecules.

The results of the transformation-driven optimization for each endpoint are described below.

### Aquatic toxicity model

In the optimization process, only significant transformations applicable to a particular molecule are used. It is also often the case that a particular transformation can be applied to a molecule in several different ways, resulting in multiple transformed molecules for each individual transformation.

Table 2 shows statistics for the aquatic toxicity optimization using matched pairs.

**Table 2:** **The number of transformed molecules generated during aquatic toxicity optimization**

**Molecule**	**Generated**	**Kept**	**Hits**	**Effectiveness**
Permethrin	1217 (27)	891 (26)	854 (24)	96% (92%)
Hexestrol	813 (10)	165 (8)	163 (8)	99% (100%)
alpha-Naphthoflavone	583 (19)	136 (13)	132 (13)	97% (100%)
Propidium	1812 (44)	1325 (37)	1316 (37)	99% (100%)
Bithionol	224 (9)	91 (8)	91 (8)	100% (100%)
Dequalinium	2095 (87)	1645 (87)	1635 (87)	99% (100%)
Oxiconazole	1211 (25)	826 (24)	798 (22)	97% (92%)
Lercanidipine	2059 (39)	1940 (39)	1928 (39)	99% (100%)

For comparison, the number of molecules generated using only the transformations based on experimental data is given in brackets.

The “generated” column gives the full number of product molecules generated using the significant transformations. The “kept” column shows the number of those molecules that passed the minimal similarity filter (50% Tanimoto similarity to the original molecule). The “hits” column gives the number of molecules for which the transformation resulted (according to the model) in a desired change of property, i.e. a reduction in aquatic toxicity. The “effectiveness” column shows the hits-to-kept ratio.

Each cell of the table contains two counts – the first represents the number of molecules obtained from both prediction-based and experiment-based transformations, while the figure in brackets gives the count for experimental-based transformations only.

It is apparent that prediction-driven transformations provide significantly more hits than experimentally-based ones. For example, prediction-based transformations provided more than 1,000 hits for the Permethrin molecule, whereas experiment-based transformations provided only 25. It is interesting that the effectiveness of predicted transformations (percentage of hits among all generated structures) is about the same as that of experimental ones.

**Figure 4:**
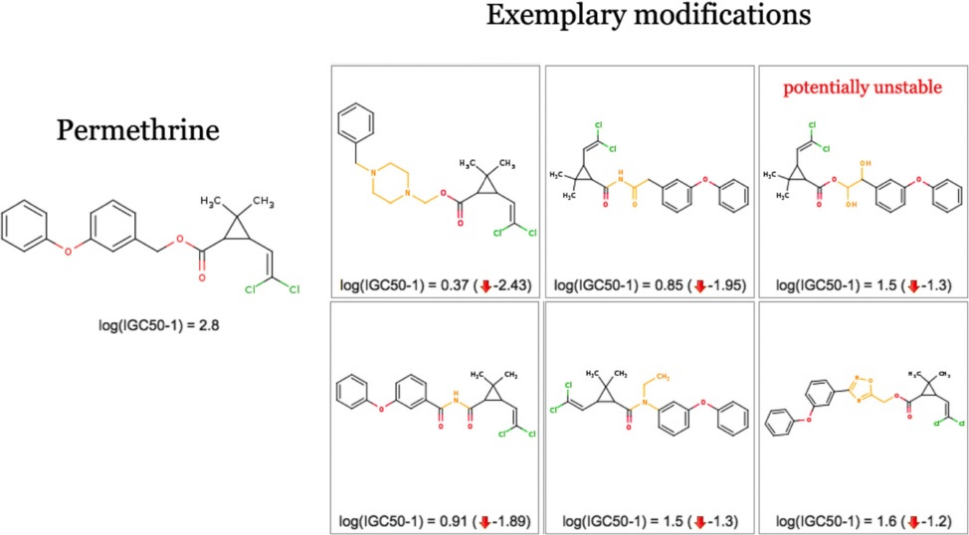
**Permethrin optimization examples.** Six exemplary modifications of Permethrin that significantly decrease its predicted aquatic toxicity (growth inhibition concentration). A decrease of 1–2 log units can be achieved by making only minor structural changes.

Figure 4 shows several exemplary modifications of the Permethrin molecule, obtained by applying significant transformations.

It should be clearly stated that, although the suggested structure modifications can be less toxic (as predicted by a QSAR model), they can be unsatisfactory in other respects: they can lose their primary effect (e.g. potency) or become chemically infeasible or unstable. One potentially unstable modification is highlighted in Figure 4.

It is also worth noting that reducing the toxicity of Permethrin, an insecticide, may not be a desired effect after all. This molecule was chosen merely to demonstrate the concept because of its high aquatic toxicity.

**Figure 5:**
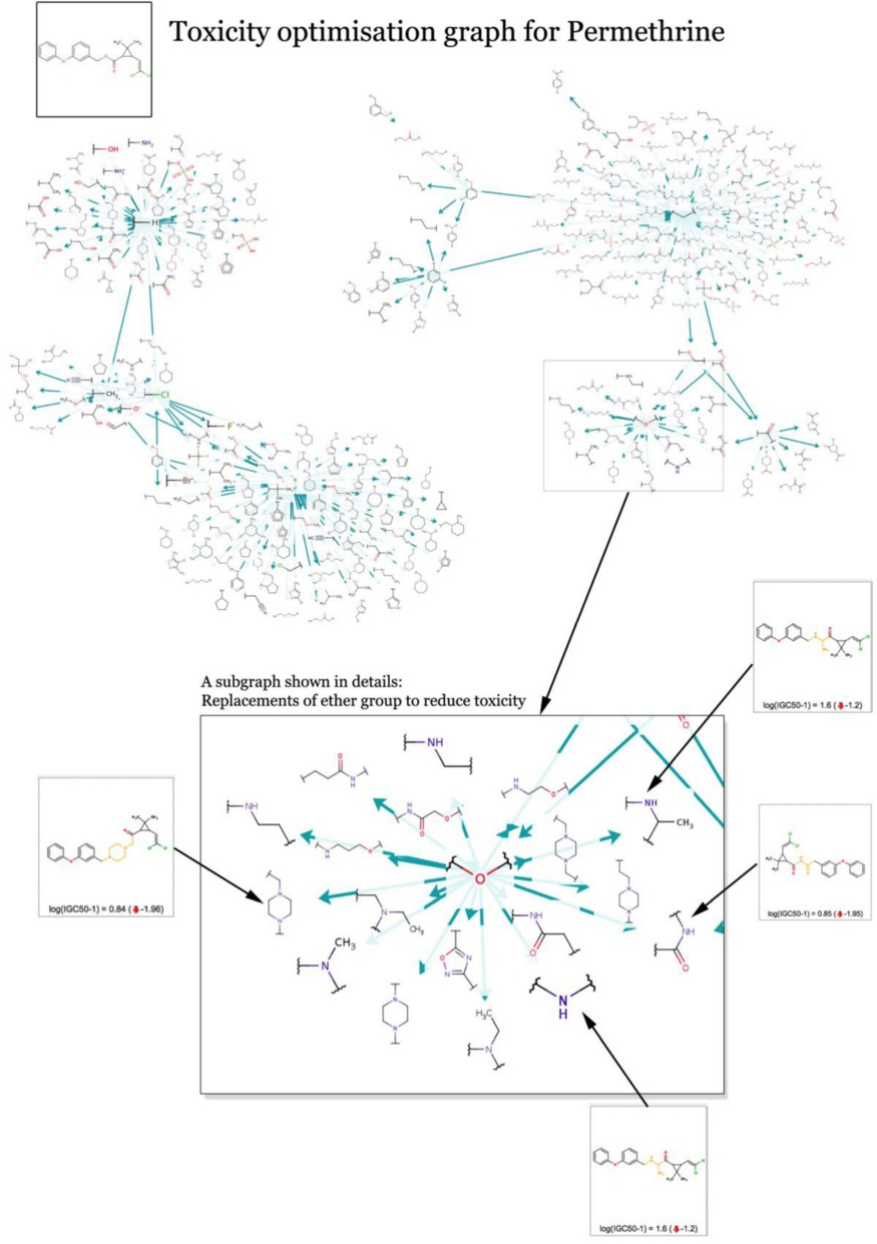
**Transformation graph for Permethrin optimization.** Graph of the transformations that affect the aquatic toxicity of the Permethrin molecule. The graph includes 393 transformations that provide replacements of several structural groups by less toxic variants. A cluster with replacements of ether groups is shown in detail together with a few examples of optimized molecules.

Figure 5 provides a visual representation of transformations applied to the Permethrin molecule. The transformations identified as reducing toxicity in Permethrin are replacements of several simple fragments or atoms to a variety of other fragments. For example, the figure highlights the replacement of an ether group by a dozen other substituents.

### Transformation amplification

**Figure 6:**
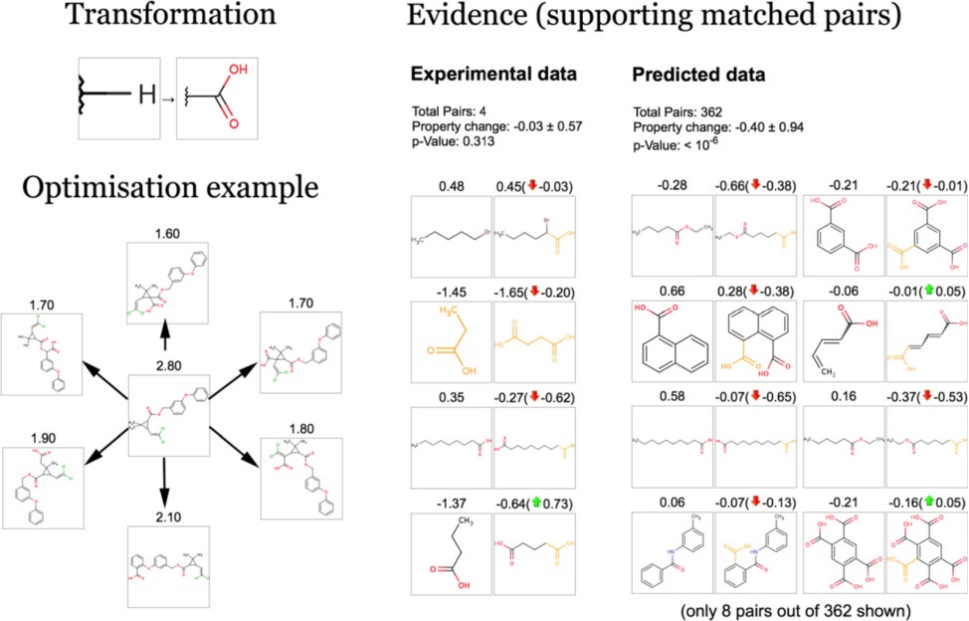
**Experimental and predicted evidence supporting the toxicity-reducing effect of a selected transformation.**

As mentioned before, these transformations are derived using predicted values. Some of the transformations were among those identified using experimental data. However, there were too few pairs to draw conclusions about the statistical significance of a toxicity-reducing effect. For example, Figure 6 shows the replacement of a hydrogen atom by a carboxyl group that has been identified as being toxicity-reducing and which has been successfully used to find non-toxic variants of Permethrin (left side of the figure). This transformation had only four experimentally measured pairs of molecules but has been “amplified” using 362 predicted pairs of molecules (p-value <10^−6^).

**Figure 7:**
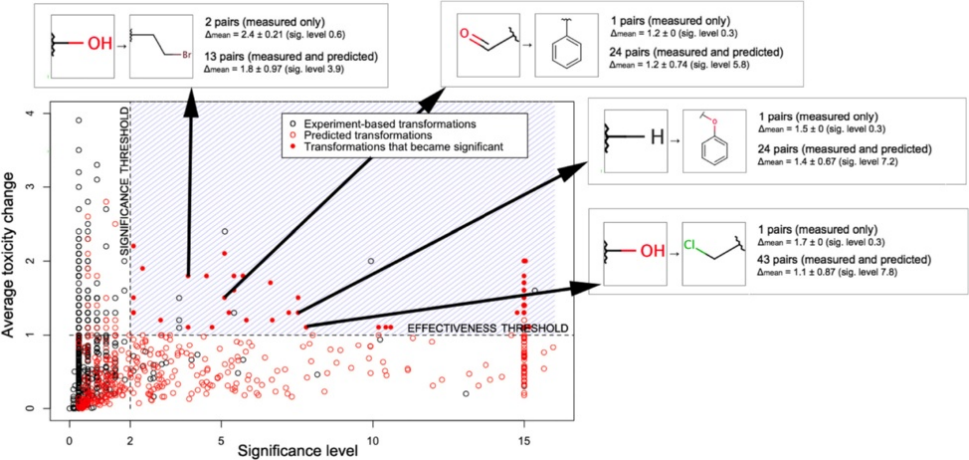
**Toxicity optimization: statistically and practically significant transformations.** The chart shows interesting transformations that are both *statistically significant* (significance level >2, p-value <0.01) and *effective* (mean toxicity change at least one log unit). A number of transformations that did not have sufficient measured pairs became significant when combined with predicted pairs (were “amplified”, shown as solid red circles).

Figure 7 gives a larger view of the “amplified” transformations. The blue dashed area shows the practically significant transformations that are both:

- effective (resulting in a toxicity change of at least 1 log unit) and
- statistically significant (p-value <0.01 or less, corresponding to a significance level of at least 2)

The black circles are the transformations based on measured data. Only a few of such transformations are “interesting”, meaning that they fall within the blue region of practical significance. The red circles show the same transformations, which have been complemented with the predicted data. The solid red circles show the “amplified” transformations that were not significant but became so after adding the predicted data.

Thus, prediction-driven MMP allows not only the discovery of new transformations, but also the amplification of existing ones by providing more evidence of the observed effect. The same phenomenon is confirmed for a classification property, CYP3A4 inhibition, in the analysis below.

### CYP3A4 inhibition model

All of the selected molecules are CYP3A4 inhibitors of different potencies. Therefore, we can use the MMP optimization process to remove the CYP inhibition activity from these molecules. Table 3 shows the results of the optimization process.

**Table 3:** **The number of product molecules generated during CYP3A4 inhibition optimization**

**Molecule**	**Generated**	**Kept**	**Hits**	**Effectiveness**
Permethrin	769 (101)	521 (86)	141 (9)	27% (10%)
Hexestrol	446 (39)	109 (11)	57 (8)	52% (73%)
alpha-Naphthoflavone	375 (64)	107 (23)	46 (7)	43% (30%)
Propidium	756 (110)	615 (96)	146 (1)	24% (1%)
Bithionol	103 (18)	39 (2)	19 (1)	49% (50%)
Dequalinium	519 (74)	487 (72)	96 (0)	20% (0%)
Oxiconazole	552 (73)	397 (47)	62 (0)	16% (0%)
Lercanidipine	1073 (148)	1037 (148)	175 (0)	17% (0%)

We can see that the experimentally based transformations yielded very few hits, with effectiveness ranging between 0% (no improvements found) and 73%. The prediction-based transformations produced significantly more hits and in most cases increased the effectiveness compared to the experiment-based transformations.

**Figure 8:**
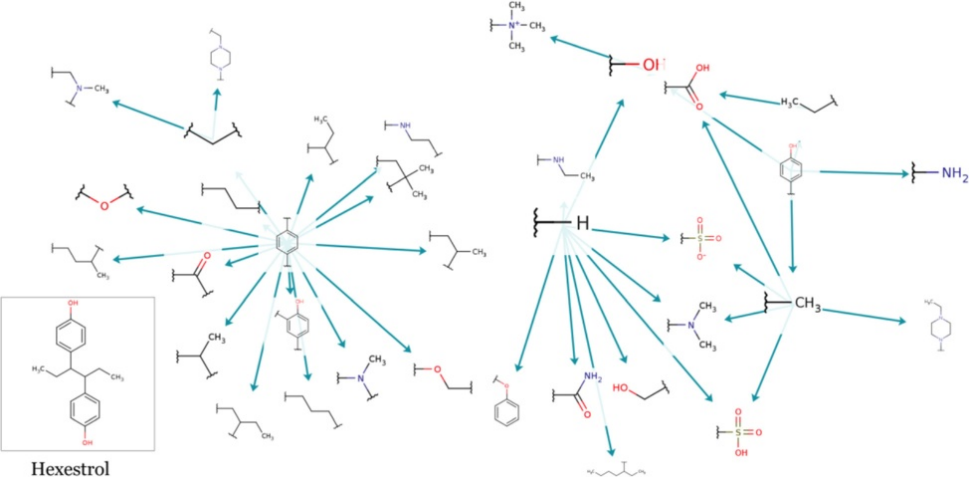
**Transformations graph of CYP3A4 optimization of Hexestrol.**

The transformations graph shown in Figure 8 gives an insight into the transformations applied to Hexestrol, which was used as an example.

We can see two clusters of transformations, which reduce the CYP3A4 inhibition activity in two different approaches.

**Figure 9:**
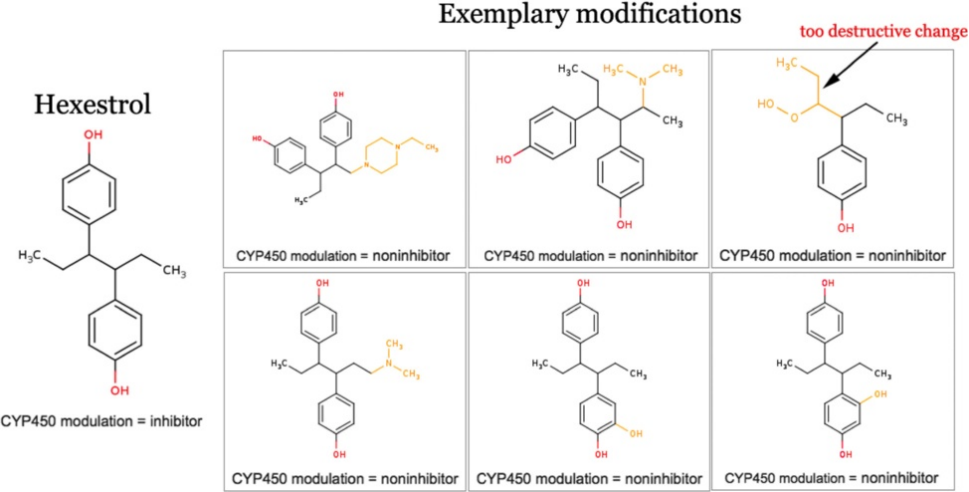
**Sample modified molecules obtained from Hexestrol after CYP3A4 inhibition optimization.** Overdestructive changes can be avoided by additional filtering by structure similarity (e.g. Tanimoto similarity).

The cluster on the left represents replacement of one of the benzene rings by a non-aromatic group, and the cluster on the right mainly represents addition of a functional group instead of a hydrogen or carbon. Figure 9 shows some of the molecules produced by the MMP optimization process using these two approaches.

Clearly, the first approach is useless in most scenarios, since it destroys the characteristic scaffold of the molecule. The resulting molecules may lose the main activity of the original molecule, which is inhibition of microtubule polymerization [12].

The second approach produces more viable molecules and in most cases tends to increase their solubility. As we can see, addition of hydroxyl groups, acetic and sulphonic acid groups and amine groups all reduce the probability of a molecule being a CYP3A4 inhibitor.

### Transformation amplification

**Figure 10:**
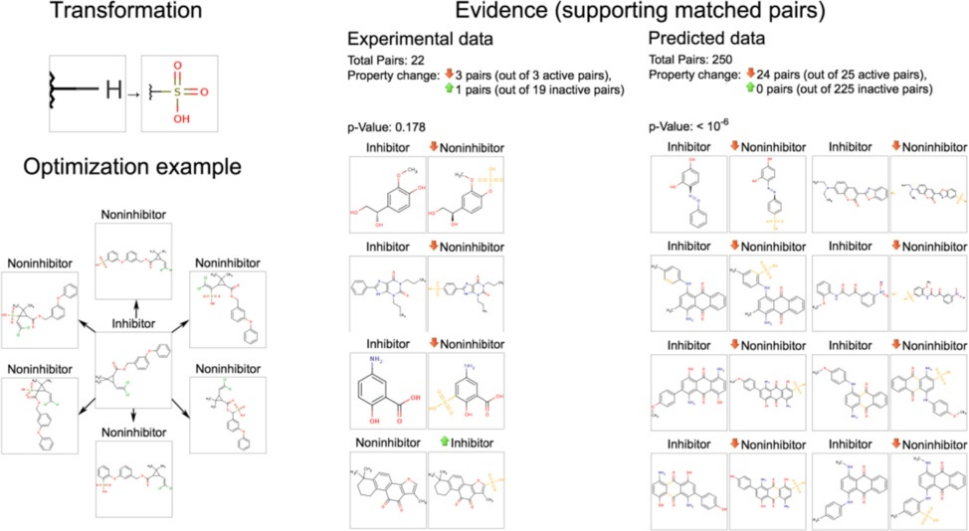
**Experimental and predicted evidence supporting the CYP inhibition-reducing effect of a selected transformation.**

Similar to the toxicity optimization example (Figure 6), Figure 10 shows that an exemplary transformation that was “inconclusive” according to the experimental data (p-value 0.18 according to 22 sample pairs) was nonetheless found to reduce CYP inhibition in a statistically significant sense according to the predicted data (p-value 0.01 according to 250 sample pairs). Thus, predictive-driven MMP analysis allows not only identification of new (predicted) transformations but also confirmation of experimentally measured ones.

Figure 10 shows that predicted pairs allow us to draw much stronger conclusions. In this example, 24 out of 25 inhibitors were “deactivated” and become non-inhibitors after applying the analysed transformation. None of the non-inhibitors became active. This shows an effect that is significant both in a statistical and a practical sense.

**Figure 11:**
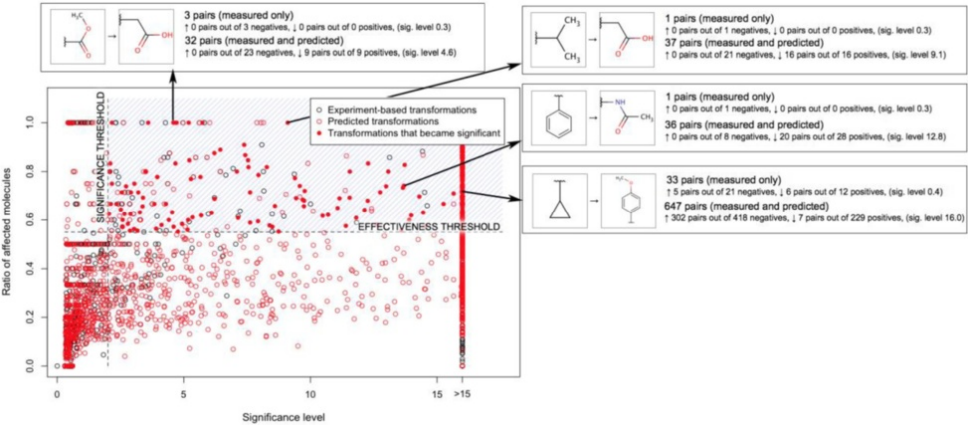
**CYP inhibition optimization: statistically and practically significant transformations.** The chart shows interesting transformations that are both *statistically significant* (significance level >2, p-value <0.01) and *effective* (ratio of deactivated molecules at least 55%). A number of transformations that did not have sufficient measured pairs became significant when combined with predicted pairs (were “amplified”, shown as solid red circles).

Similar to the toxicity use case, there are a number of “amplified” transformations that were identified as both statistically and practically significant after consideration of predicted pairs. Such transformations are shown as solid red circles in Figure 11.

The “amplified” transformations are not identified using *exclusively* the predicted data. Besides the predicted pairs, there are “real”, experimentally measured pairs associated with such transformations, which makes them more credible for a medicinal chemist.

The two use cases described above show that prediction-driven MMP analysis allows the identification of transformations that affect particular molecular properties, so-called significant transformations. Moreover, such analyses identify more transformations than classic experiment-based MMP analysis. This allows the molecule optimization process to be improved – more transformations result in more optimized structural suggestions.

The transformation graphs appeared to be a useful tool for visualization of hundreds of transformations and facilitating their interpretation. Thus, the graphs in Figure 4 and Figure 8 allowed identifying the fragments that tend to induce a particular activity (CYP inhibition, aquatic toxicity or mutagenicity).

The detected MMP transformations can be also exported and used in external applications, such as Molpher [13], to optimize new chemical structures. They can be also used as a part of discovery tools, such as the Self Organizing Hypothesis Network (SOHN) [14], by enhancing the tool with clearly interpretable knowledge units.

Several important points should be noted regarding the limitations of transformation-driven molecular optimization. We discuss these limitations and suggest possible solutions below.

### Chemical context of transformations

**Figure 12:**
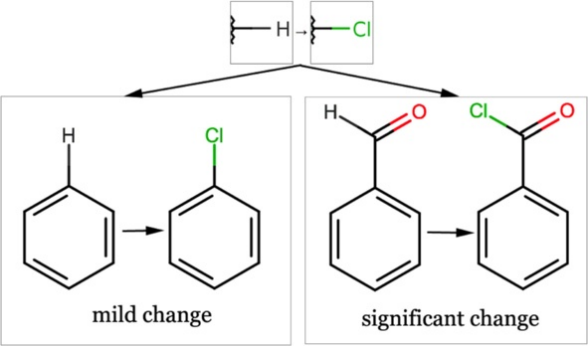
**Importance of chemical context for transformations.** The same transformation can have significantly different effects in different contexts.

The effect of a particular molecular transformation can depend significantly on the context of the change. For example, as illustrated in Figure 12, replacement of a hydrogen atom by chlorine could have different effects depending on whether it is connected to an aromatic ring or part of a reactive group.

The current implementation of MMP analysis ignores the surrounding of the point of change and “averages” the effects of a transformation for all possible contexts. Although we have shown that such an approach gives satisfactory results, it is suboptimal and, generally speaking, it makes sense to distinguish transformations in different contexts. One approach is to prohibit the storing of “small” transformations (e.g. by restricting the minimum atom count in the fragment) and to accept only the larger ones, which include more context information. Furthermore, expert knowledge in the form of a rule set can be employed. Such sets of rules can, for instance, prevent indexing of transformations that would destroy chemical groups or aromatic ring systems.

Finally, the context problem can be addressed by using chemistry-aware fragmentation algorithms, such as RECAP [15]. Such fragmentation takes into account functional groups; this makes it possible for example to distinguish an -OH group (alcohol) and -OH as a part of a carboxylic acid. Thus, the carboxyl group would be treated as a whole during the MMP identification process.

In summary, the aforementioned approaches require an elaborate definition of “acceptable” transformations and improved fragmentation techniques. Such enhanced indexing of molecular pairs could be a future improvement of the methodology suggested here.

### False positives

Not all “significant” transformations affect the molecule in a desired way. For example, transformations can decrease toxicity for most molecules but increase it for some fraction of the data. This is the reason why the “transformation effectiveness” (as specified in Tables 3 and 4) can be significantly less than 100%. Furthermore, false positives may result from the multiple comparisons problem, which is partially corrected by the Holm-Bonferroni method. In either case, the problem of false positives is not crucial, since the molecules without predicted improvements are explicitly filtered out.

**Table 4:** **Summary of the models used for prediction-driven MMP analysis**

**Endpoint/Property**	**Training set size**	**Web-link**
Aquatic toxicity	1 093	http://ochem.eu/model/3
CYP3A4 inhibition	15 316	http://ochem.eu/model/163

### Predictions

We use QSAR models both to identify transformations and to evaluate whether they have the desired effect on new chemicals. The predictions given by QSAR models can be inaccurate, even considering the applicability domain estimates. This issue applies mostly to the purely prediction-driven transformations that do not have sufficient experimental evidence to confirm the predicted effect (so-called “amplified” transformations). This issue can be addressed either by gathering more experimental data to confirm/discard the transformation effect or by alerting users to the fact that the transformation is based on predicted data. While implementation of the first approach is open-ended, the latter approach is already integrated into OCHEM. Namely, each transformation has a profile page, where users can see whether the transformation is based on experimental data, predicted data or both.

Importantly, OCHEM comes with integrated applicability domain assessment and can estimate the accuracy of each prediction individually. Thus, the user is warned of the risks and can choose to ignore potentially unreliable predictions.

### Lost potency

The optimized structures may not possess useful properties of the original molecule. Thus, we may make a compound less toxic but decrease its bioavailability or lose its potency (e.g. binding affinity) altogether. This problem can be addressed by multi-criterial optimization (e.g. using the transformations that reduce toxicity but do not affect lipophilicity) or, more universally, by post-filtering the optimized molecules by running QSAR models or estimating their binding affinity using docking-derived techniques.

### Chemical feasibility and stability

The suggested modifications can be unstable or difficult (or infeasible) to synthesize. Assessment of feasibility and stability can be incorporated as an additional filter to eliminate such structures. There are already many programs, such as SYLVIA (http://www.molecular-networks.com/products/sylvia) by Molecular Networks or REACTOR (http://www.chemaxon.com/products/reactor/) by ChemAxon that can be used for such purposes.

The assessment of feasibility is a different topic. In general, chemical modifications and therefore reactions can be described as feasible if they occur spontaneously without an external source of energy. Such reactions are therefore thermodynamically favourable. Hence, it is common practice to filter such reactions using a set of fixed rules that are meaningful from the synthesis point of view [16]. There are a handful of computational tools that identify these chemical sensible rules in a Retro-synthetic analysis [17,18].

OCHEM was developed with flexibility and modularity in mind. This is reflected in the ease of integration of third-party utilities. Therefore, the tools for filtering out unstable or infeasible compounds can be added as one of the independent steps of the structure-optimization process.

### Complementing approaches

MMP analysis can be complemented by other interpretation techniques. First, similarity maps [19] can visualize differences between molecular structures. Such visualization is not restricted to single-point changes as with MMPs. Second, feature networks [20] can be used to interpret individual predictions by identifying the activating and deactivating structural features. Finally, approaches that estimate the contributions of individual fragments to the activity of interest [21] can be helpful in confirming “significant” transformations. Integrating such utilities into OCHEM could allow users to obtain a comprehensive interpretation of predictions.

To summarize, if complemented by additional filtering steps, prediction-driven MMPs and transformations are useful for hit-to-lead optimization. Their public availability will contribute to the widespread use of the computational chemistry [22] tools on the Web [23].

## Conclusions

In this study, we investigated pairs of molecules that have only minor localized differences in their structures – so-called matched molecular pairs (MMPs). We suggested a new concept of *prediction-driven MMPs*, utilizing predictions given by QSARs for large chemical libraries to generate simple transformation rules that affect the activity of interest (so-called “significant” transformations).

We saw clearly that such an approach generates additional knowledge compared to classical MMP analysis. We showed that, compared to traditional MMPs, which are derived from experimental data only, predicted-driven MMPs provide added value and could be used to guide the molecular optimization process by generating many more suggestions for medicinal chemists.

Prediction accuracy and reliability were addressed by incorporating the applicability domain of QSAR models and the estimated prediction accuracy for each analysed molecule.

The usefulness of the described methodology was exemplified by two practical use cases. Two endpoints were analysed: aquatic toxicity and CYP inhibition potential. For both endpoints and their respective datasets, we identified all MMPs and their respective molecular transformations. A particular focus was on the identification of “significant” transformations that reduced toxicity or CYP inhibition potential. These transformations were identified using both experimental data and predicted data obtained using QSAR models applied to two large chemical datasets with more than 400,000 compounds. We showed that predicted data enabled identification of a large number of significant transformations and amplified the otherwise insignificant transformations useful in the molecule optimization process.

The approach developed here has limitations and potential for improvement. To convert the suggested methodology into a tool that could be used by medicinal chemists, a number of questions still need to be answered. These include: “How can we ensure that the modified compounds are chemically feasible and stable? How can we take the chemical context of the transformations into account? And how can we ensure that the suggested structures do not lose the desired properties of the original compounds (e.g. potency or drug-likeness)?” The article included a discussion on the required improvements.

Importantly, all the methodological developments presented in this article have been implemented as a software platform, which includes identification of MMPs, extraction of rules (significant transformations), and integration with an online QSAR framework and chemical database (OCHEM). The “tip of the iceberg” is the molecular optimization utility “MolOptimiser” which, with a couple of mouse clicks, allows medicinal chemists to optimize their molecules online using the knowledge extracted from predictions of half a million compounds by a dozen models.

The current implementation comes with a transformations database for a number of endpoints, such as mutagenicity, aquatic toxicity, lipophilicity, solubility, melting point and CYP inhibition. However, the database of rules is expandable: users can upload their own datasets, build QSAR models, identify significant transformations and save them for further use.

Prediction-driven MMP analysis will help to open the “black boxes” of QSARs, to interpret the models and to facilitate their practical application in *in silico* drug design.

## Methods

### Basic definitions

#### Matched molecular pair

In a broad sense, an MMP is defined as a pair of molecules that differ by a minor single point change only. The “minor single point” must be defined in specific technical terms. In this study, we will consider a pair of molecules a *matched pair* if the differing fragment is less than 10 atoms in size and has fewer atoms than the unaffected part of either molecule.

### Molecular transformation

**Figure 13:**
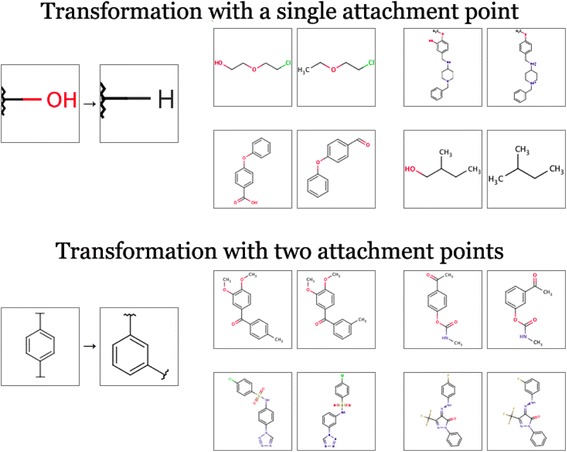
**Exemplary molecular transformations.** Single and double-point transformations shown.

Each molecular pair is associated with a particular transformation. An example transformation is the replacement of one functional group by another. More specifically, we define a transformation as a replacement of a molecular fragment having one, two or three attachment points by another fragment. Figure 13 shows two examples of molecular transformations with four corresponding molecular pairs each.

### “Significant” transformations

One of the main ideas of MMP analysis is that some molecular transformations tend to systematically affect particular molecular properties. For example, a transformation may systematically decrease toxicity or increase lipophilicity of chemical compounds. We will label transformations that affect a particular property/activity in a statistically significant sense *significant transformations*.

A transformation is considered significant if it increases the property value “more often” than it decreases it, or vice versa. Thus, the distribution of increasing and decreasing pairs should be significantly different from the binomial (“no effect”) distribution with a particular p-value (usually *0.05*). More specifically, the p-value can be calculated using Formula 1:

1 $$ pValu{e}_{\mathrm{regression}} = Prob\ \left\{\xi \right.\ \left( 0.5,\ N\right)\le\ min\kern0.5em \left({n}_{pos},\ {n}_{\mathrm{neg}}\kern0.5em \right)\Big\} $$

where *Prob* stands for probability, *n*_*pos*_ and *n*_neg_ are the number of pairs that decreased and increased the property, *N* is the total number of pairs, and *ξ* (0.5, *N* )is the binomial distribution with *N* trials and a probability of *0.5*.

The mechanics of “statistical significance” are somewhat different for binary classification properties, where a compound is classified as either “active” or “inactive”. Such properties include, for example, mutagenicity or CYP inhibition. For binary classification problems, we consider the transformation as significant if the percentage of “active” molecules in the analysed set is significantly changed after the transformation. Technically, we define the binomial distribution of active as our “null hypothesis” and calculate the p-value as follows:

2 $$ pValu{e}_{classification}= min\ \left(\  Prob\ \left\{\xi \left(\frac{n_{pos}}{N},\ N\right)\ \le {\tilde{n}}_{pos}\ \right\},\  Prob\ \left\{\ \xi\ \left(\frac{n_{{}_{\mathrm{neg}}}}{N},\ N\right)\ \le {\tilde{n}}_{\mathrm{neg}}\ \right\}\ \right) $$

where *n*_*pos*_, *n*_neg_, *ñ*_*pos*_*and ñ*_neg_ are the number of “active” and “inactive” molecules in the sets before and after applying the transformation.

**Figure 14:**
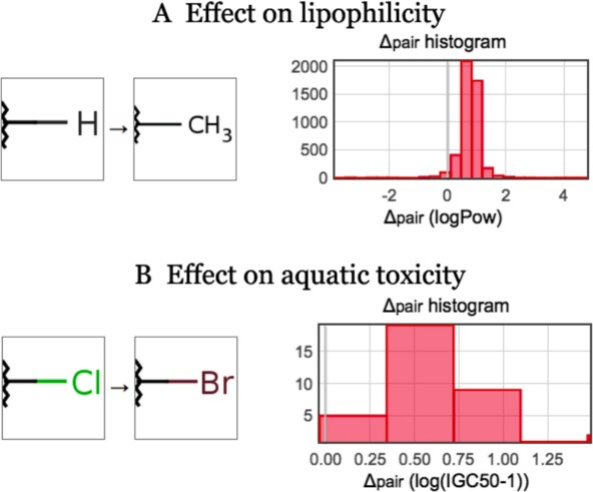
**Effect of a transformation on molecular properties. A)** A simple transformation and the distribution of its effect on the octanol/water partition coefficient. The histogram is visually biased to positive values: on average, this transformation increases lipophilicity. **B)** Replacement of carbon by bromine significantly increases aquatic toxicity.

Visually, significant transformations can be described by distributions of “pair deltas”, that is, by the difference of the property values between the molecules in a matched pair. Figure 14 shows a histogram of pair deltas for the octanol/water partition coefficient and aquatic toxicity for two simple transformations. The delta-pair histograms were built using the data publicly available in the OCHEM database.

Sometimes it is more convenient to use the significance level, which is a log-scale representation of the p-value calculated according to Formula 3. Thus, a p-value of *0.01* corresponds to a significance level of “*2”*, while a p-value of *0.001* corresponds to a significance level of “*3”* and so on.

3 $$ significance\_\  level=- \log {}_{10}\ (pValue) $$

As many transformations are analysed, some transformations can pass the p-value threshold and be misclassified as “significant” by mere chance. This phenomenon, known as the “multiple comparisons” problem, can be partially addressed using the Holm-Bonferroni method [8].

### Practical significance

In practice, it is important that the transformation effect is not only statistically significant but also practically significant in absolute terms. A transformation may be statistically significant but lead to a low absolute effect (e.g. a toxicity reduction of 0.1 log unit). The practical significance is defined for each endpoint individually and is subjective.

For the purposes of this study, we will ignore the practical significance at the transformation identification stage. Instead, we will consider it when using these transformations in the molecular optimization process, which is often the ultimate goal of the MMP analysis.

### MMPs in the QSAR context

Here we describe a number of analytical methods that allow us to interpret QSAR models using the MMP approach.

### Predicted values instead of experimental data

In the MMP-related scientific literature, statistical analysis is usually performed using experimental data. One of the major contributions of the current study is to use predicted values in addition to experimental ones. This approach gives two important advantages.

First, experimental data are often limited and do not have sufficient measurements for a meaningful MMP-based statistical analysis. Using predictions for large datasets (e.g. compound libraries) allows us to overcome the data limitation problem.

Second, using predicted values allows us to extract the rules, based on the model’s point of view. We do not directly analyse the data, rather we view it through the lens of the model. This allows the analyst to interpret the model itself rather than interpreting the available experimental data.

### Applicability domain

For any analysis based on predictions, it is of crucial importance to consider the reliability of predictions and to take into account the applicability domain (AD) of the models [24,25]. The applicability domain of QSAR models is a research field in itself and has received abundant attention in the literature [26-28]. For this study, we used the standard deviation (STD) of ensemble predictions to define the applicability domain. This was shown to be the most reliable approach for differentiating accurate and inaccurate predictions and for estimating the prediction accuracy (the root mean square error, RMSE, for classification models or the correct prediction rate for classification models), as described elsewhere [27,28].

The prediction accuracy is taken into account during identification of “significant” transformations. Technically, we generate 1,000 replicas of the analysed dataset by perturbing each prediction with an amount of Gaussian noise with a magnitude (standard deviation) depending on the estimated accuracy of the prediction. Such a bootstrapping process is intended to exclude transformations that are based on non-reliable predictions.

### Transformations graph

**Figure 15:**
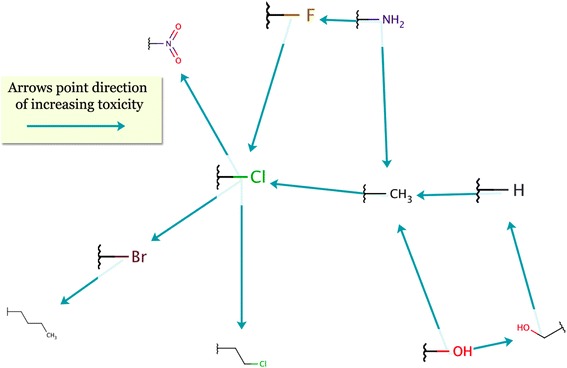
**A transformations graph for aquatic toxicity.** Arrows point towards the direction of increasing toxicity. For example, it can be seen that the presence of bromine is potentially more toxic than the presence of chlorine, whereas the hydroxyl group is the least toxic residual in this example.

Each molecular transformation is a replacement of one molecular fragment by another; that is, a transformation is a relation between two molecular fragments. Based on the “significant” transformations, it is possible to create a directed graph of molecular fragments. Each node in the graph is a fragment, and each edge a significant transformation. Such graphs can display a number of transformations and enable better interpretation. The graph in Figure 15 shows a part of the transformations related to Aquatic toxicity.

Compared to a simple list of significant transformations, such a graph provides a more visual and interpretable insight.

### Delta-pair chart

**Figure 16:**
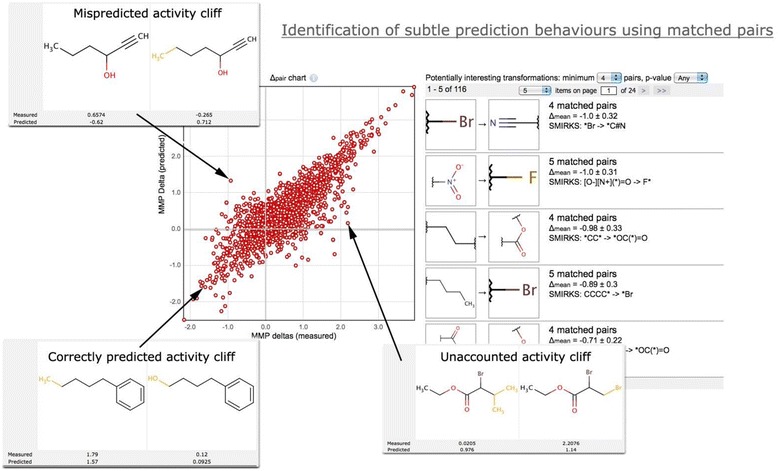
**A delta-pair chart for an aquatic toxicity model.** Three representative cases of activity cliffs are shown. The right part shows the significant transformations for aquatic toxicity.

MMPs can be used to identify subtle prediction behaviour, e.g. model reaction to activity cliffs. We define a chart that shows the *actual* and *predicted* effects of molecular transformations a *delta-pair chart*. Such charts can identify pairs that have a significant activity change, referred to as activity cliffs. The chart in Figure 16 shows the unaccounted and mispredicted activity cliffs, which can help identify “weak points” in a model. The screenshot is based on an aquatic toxicity model [27] publicly available at https://ochem.eu/model/3.

The points on the diagonal correspond to a perfect match between the experimental and predicted effects of a molecular transformation. Conversely, the points in quadrants 2 and 4, (−, +) and (+, −), correspond to mispredicted activity cliffs. The larger number of points in quadrants 1 and 3 indicates that the model has correctly learned the majority of structure activity relationships presented in the data.

Since the direction of an MMP transformation can be arbitrarily changed, the position of points on the plot can easily be flipped between quadrants 1 and 4 and between quadrants 2 and 3.

### Datasets and models

For the identification of matched pairs and molecular transformations we used two chemical libraries, ChemDiv and EINECS, described below.

### ChemDiv compound library

Chemical Diversity (ChemDiv) is a chemical provider and contract research organization. It maintains and develops several general purpose and targeted molecular libraries for a variety of applications. In this study, we used a chemically diverse ChemDiv library containing 391,145 molecules.

### EINECS compound library

The EINECS (European INventory of Existing Commercial chemical Substances) dataset comprises 68,779 unique chemical compounds that are produced in or imported to Europe in amounts of more than one ton per year. These compounds are intended for the registration in REACH program and, therefore, they are of particular interest for the assessment of their environmental hazard.

To demonstrate the concept and methodology of prediction-driven MMPs, we used the following two QSAR/QSPR models, described below and summarized in Table 4.

### Aquatic toxicity model

This model [27] predicted the growth-inhibition concentrations measured on a ciliated protozoan Tetrahymena *pyriformis*. This is an established screening tool for toxicity. The model was developed using E-state indices and Associative Neural Networks [29] and produced one of the highest accuracies in a benchmarking study to predict environmental toxicity [30].

### CYP3A4 model

The CYP model [31] was developed for inhibition of Cytochromes P450 (CYP), a superfamily of enzymes involved in the metabolism of a large number of xenobiotic compounds [32,33]. Over 75% of currently marketed drugs are cleared with the help of CYP enzymes, and almost half of these are metabolized by the CYP3A4 enzyme [34]. Inhibition of CYP3A4 may therefore lead to toxicity by drug-drug interaction. This makes prediction of CYP3A4 enzyme inhibition one of the main goals in early stage drug discovery.

In this study, we use a classification model, which assigns “inhibitor” and “non-inhibitor” labels to the predicted compounds. It was developed using a training set of over 15,000 compounds obtained by high-throughput screening [35]. The model was built using E-state indices and ALogPS descriptors using Weka implementation of J48 decision trees. Additionally, stratified bagging was used to handle the imbalance between active and inactive molecules in the training set. The resulting model has one of the highest published accuracies for comparable datasets [31].

### Implementation aspects

#### Integration with OCHEM

All aforementioned MMP utilities have been tightly integrated with the Online Chemical Modelling Environment (OCHEM, available at http://ochem.eu). OCHEM is an online platform that allows scientists to perform the full cycle of QSAR research, including:

- data collection, upload and management
- development of regression and classification QSAR models with a dozen machine learning methods
- integration with more than 20 molecular descriptor packages (both free and commercial)
- applicability domain assessment
- running predictions on published models for a number of endpoints (Ames test, CYP inhibition, Aquatic toxicity, Melting and Boiling points, Bio-concentration factor, Fish toxicity and many more)
- a database of structural alerts with a screening utility (the ToxAlerts module) [36]

A full list of OCHEM features can be found in the literature [22] or in the knowledge base [37].

The MMP utilities are tightly integrated with the OCHEM user interface. The utilities can be summarized briefly as follows: analysing MMPs for a dataset, identification and saving significant transformations, constructing a delta-pair chart for a model, drawing molecular fragment transformation graphs, and searching for transformations that affect multiple properties in a desired manner (“transformation optimizer” utility). More detailed documentation and a user guide to MMPs in OCHEM can be found online in the knowledge base [22,37].

### Automatic indexation of MMPs

All molecules stored in OCHEM are automatically screened for identification of MMPs. Technically, an asynchronous background job fragments all the molecules and creates an index as described in [6]. The fragmentation is performed on a distributed calculation system provided by OCHEM. A second job uses this index to identify MMPs, and create and update molecular transformations. OCHEM tracks the uniqueness of molecules using InChi hash-keys [38,39] in order that each unique molecule is processed only once.

An important consequence of the asynchronous indexing procedure is that new molecules are not available immediately for MMP analysis but only after indexing, which is usually a matter of a few hours.

**Figure 17:**
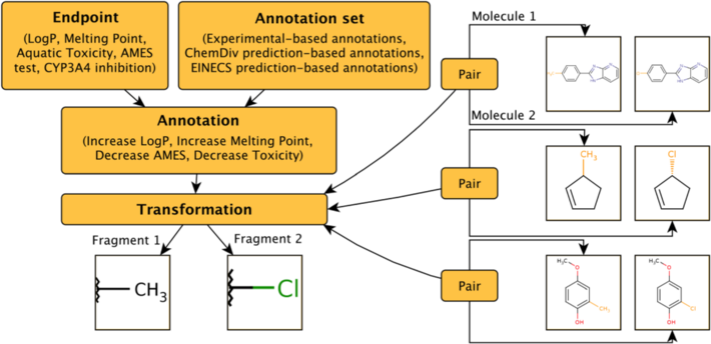
**A simplified database schema to store MMPs, transformations and transformation annotations.**

A simplified database schema for storing MMPs and transformations is shown in Figure 17.

### Technical limitations

To deal with the combinatorial explosion problem, only molecules with 40 or fewer breakable bonds are considered for MMP indexing. Larger molecules are ignored. The variable part of the molecule should have no more than 10 atoms and fewer atoms than the main scaffold of the molecule.

### Technical statistics

Currently, the OCHEM database contains 700,000 indexed molecules, corresponding to about 12 million matched molecular pairs categorized in ~500,000 unique molecular transformations. The total size of the database (excluding molecular structures) is 3 gigabytes.

## Abbreviations

CYP: Cytochrome P450
EINECS: European INventory of Existing Commercial chemical Substances
MMP: Matched Molecular Pair
MMPA: Matched Molecular Pairs Analysis
OCHEM: On-line CHEmical Modeling environment, http://ochem.eu
QSAR: Quantitative Structure-Activity Relationship
